# Transcriptional regulation of Bcl-2 gene by the PR/SET domain family member PRDM10

**DOI:** 10.7717/peerj.6941

**Published:** 2019-05-15

**Authors:** Na Chen, Taobo Hu, Yuanyuan Gui, Jieying Gao, Zhihong Li, Shi Huang

**Affiliations:** 1 Center for Medical Genetics, School of Life Sciences, Central South University, Changsha, Hunan, China; 2 Department of Orthopedics, The Second Xiangya Hospital, Central South University, Changsha, Hunan, China

**Keywords:** PRDM10 protein, Bcl-2, B-cell lymphoma-2, Transcription, The Cancer Genome Atlas

## Abstract

Bcl-2 (B-cell lymphoma 2) protein is localized in the outer membrane of mitochondria, where it plays an important role in promoting cellular survival and inhibiting the actions of pro-apoptotic proteins. PRDM10 is a member of the PR/SET family of epigenetic regulators and may play a role in development and cell differentiation. Here we show that human PRDM10 contributes to the transcriptional regulation of human Bcl-2 gene. We found that PRDM10-depletion in human cells reduced the expression of Bcl-2 protein and over-expression of PRDM10 promoted Bcl-2 protein expression. Furthermore, luciferase reporter activity of Bcl-2 gene P1 promoter was significantly increased in cells co-transfected with PRDM10, and PRDM10 was able to bind to the Bcl-2 P1 promoter *in vivo*. Using The Cancer Genome Atlas (TCGA) data set, we found weak positive correlation between PRDM10 and Bcl-2 in several cancer types including cancers of the breast, colon, and lung tissues. These data identify a novel function for PRDM10 protein and provide insights on the transcriptional control of Bcl-2 expression.

## Introduction

The B cell lymphoma-2 (Bcl-2) protein belongs to the Bcl-2 protein family and plays an important role in the regulation of programmed cell death or apoptosis (Chao & Korsmeyer, 1998; Letai et al., 2002). Bcl-2 over-expression has been observed in a wide variety of cancer (Aird et al., 2019; De Veirman et al., 2018; Wu et al., 2001; Martin & Dowsett, 2013; Reed et al., 1994). In addition, the increased cell survival due to overexpression of Bcl-2 has been shown to contribute to tumor development and resistance to various anti-cancer therapies (Reed et al., 1994; Inoue-Yamauchi et al., 2017; Delbridge et al., 2016). Thus, targeting the anti-apoptotic Bcl-2 proteins represents a promising strategy for the treatment of cancer (Davids & Letai, 2012). The Bcl-2 selective inhibitor ABT-199 (Venetoclax) has excellent anti-leukemia activity against chronic lymphocytic leukemia and was approved by the FDA in April 2016 (Souers et al., 2013; Pan et al., 2014; Touzeau et al., 2014; Vaillant et al., 2013). It has proven to be an effective approach to inhibit the interaction of Bcl-2 and BH3-only proteins by small molecules (Oltersdorf et al., 2005; Tse et al., 2008). However, the nature of dynamic protein-protein interactions and acquired resistance poses a challenge to Bcl-2 inhibitors (Zinzalla & Thurston, 2009; Choudhary et al., 2015; Juin et al., 2013). It is conceivable that targeting regulation of Bcl-2 at the transcriptional level may provide an alternative strategy for cancer therapy. Better understanding of Bcl-2 biology may have important clinical significance for better treatment of cancer.

Two promoters mediate transcriptional control of the Bcl-2 gene (Seto et al., 1988). The 5′ promoter (P1) is located 1,386 to 1,432 bp upstream of the Bcl-2 translation start site, and it is a TATA-less, GC rich promoter with multiple transcription start sites (−1,394, −1,399, −1,406, −1,410, and −1,432) and positioned in proximity to a nuclease hypersensitive site (Young & Korsmeyer, 1993). The start sites of the 3′ promoter (P2) are located 1.3 kb downstream of the P1 promoter (Onel et al., 2016). The P1 promoter is the major driver of Bcl-2 expression (Catz & Johnson, 2001; Onel et al., 2016). Several genes have been identified that regulate P1 promoter either directly or indirectly, for example, the tumor suppressor gene product p53 (Miyashita & Reed, 1995), the HIV-1 Tat protein (Zauli et al., 1995), the G-quadruplex Pu39 and P1G4 (Dai et al., 2006; Onel et al., 2016). The nuclear factor kB (Catz & Johnson, 2001) and the POU family member Brn-3a (Smith et al., 1998) have been shown to regulate the P2 promoter transcription. However, the regulation of Bcl-2 gene expression remains to be fully understood.

The PRDM10 protein belongs to the PRDM family of proteins that contain the PR domain (PRDI-BF1-RIZ1 homology domain) shared by many histone lysine methyltransferases (Huang, 2002; Mzoughi et al., 2016; Buyse, Shao & Huang, 1995; Huang, Shao & Liu, 1998). The expression pattern of PRDM10 indicates a potential role in mouse embryonic development including somite and craniofacial formation (Park & Kim, 2010; Park et al., 2013), and in corneal endothelial cell differentiation and proliferation (Rolev et al., 2017). In addition, PRDM10 protein is over-expressed in liver cancers and non-alcoholic fatty liver disease (Rolev et al., 2017; Zhang et al., 2018; Abdollahi, Zamanian & Hatami, 2017), indicating a role in facilitating tumorigenesis. Here, we studied whether PRDM10 protein may promote the action of oncogenes such as Bcl-2 gene.

## Material and Methods

### Cell culture

HEK293, MCF-7B, and Hela cell line purchased from the American Type Culture Collection-ATCC (Manassas, VA, USA). HEK293 and Hela cells were cultured in Dulbecco’s modified Eagle medium (DMEM) supplemented with 10% (v/v) fetal bovine serum (FBS, GE Healthcare Life Sciences), 4 mM L-glutamine, 4,500 mg/l glucose at 37 °C under a atmosphere of 5% CO_2_. MCF-7B cells were cultured in Roswell Park Memorial Institute (RPMI) including 10% FBS at 4 mM L-glutamine, 4,500 mg/l glucose at 37 °C under an atmosphere of 5% CO_2_.

### Plasmid constructs

The flag tagged full length PRDM10 expression plasmid PRDM10-pCMV4A-Flag was constructed by inserting full length PRDM10 cDNA from a human cDNA library into the vector pCMV4A. The Bcl-2 P1 and p2 promoter-luciferase construct has been described previously (Duan, Heckman & Boxer, 2005). The Bcl-2 P1 promoter reporter plasmid was generated by inserting the (−1,386 to −1,444 bp) human Bcl-2 P1 promoter sequence in front of the firefly luciferase reporter gene as in pGL3 basic vector. The Bcl-2 p2 promoter construct was created by inserting the (−754 to +1 bp) human P2 promoter sequence (Catz & Johnson, 2001)

### Transfection experiments

PRDM10-specific depletion was performed by transfecting 2 × 10^6^ cells with 3ug of PRDM10-specific siRNA or Control-siRNA using Lipofectamine® 2000 (Thermo Fisher Scientific, Waltham, MA, USA) according to the manufacturer’s instruction. Cells were harvested 48 h after the RNAi treatment. The siRNA duplexes were designed as 21-mers with 3′-dTdT overhangs and synthesized by GenePharma (Shanghai, China). PRDM10-siRNAs were directed against the PRDM10 sequence. PRDM10-siRNA-1(+): 5′-GCUCUACAUAGACAGGU UUTT- 3′ and PRDM10-siRNA-1(−): 5′-UUAUGAACUGGCAAUGAGGTT- 3′; PRDM10-siRNA-2(+): 5′-CCUCAUUGCCAGUUCAUAATT- 3′ and PRDM10-siRNA-2(−): 5′-UUA UGAACUGGCAAUGAGGTT- 3′; Control-siRNAs are against the sequence of luciferase:Con-siRNA(+): 5′-UUCUCCGAACGUGUCACGUTT- 3′ Con-siRNA (−): 5′-A CGUGACACGUUCGGAGAATT- 3′. For the overexpression experiments, 2 × 10^6^ cells were transfected with 3 ug of PRDM10-pCMV4A or control plasmids by using Lipofectamine® 2000. PRDM10-siRNA, control-treated and PRDM10-pCMV4A treated cells were analyzed by Western blotting.

### Western blot analysis

Cell lysates were prepared by incubating for 30 min in a lysis buffer containing 25 mM Tris (pH 7.5), 75 mMNaCl, 5% glycerol, 2% SDS and protease/phosphatase inhibitors (Sigma) followed by centrifuging at 10,000 g for 10 min at room temperature. The protein concentration was measured using the BCA protein assay kit (Thermo Fisher Scientific). Proteins were separated by SDS-PAGE and transferred electrophoretically onto polyvinylidene fuoride (PVDF) membranes (Millipore), which were incubated overnight with the antibodies as described under Table S1. The bands were detected by the MiniChemi™ Chemiluminescence imaging system (Sage Creation, Beijing, China).

### Total RNA isolation and quantitative RT-PCR

Total RNA was isolated using Trizol (Invitrogen) following the manufacturer’s instructions. Reverse transcription of RNA was performed using the ThermoScript RT-PCR System (Invitrogen). Quantitative PCR amplification was performed on the Bio-Rad iCycler-iQ system (Bio-Rad, Hercules, CA,USA) using the iQ-SYBR Green Supermix (Bio-Rad). Primers are described under Table S2.

### Transfection and luciferase assay

HEK293 cells (8 × 10^4^) were cultured in 500 µl DMEM including 10% FBS overnight using a 24 well plate at 37 °C, 5% CO_2_. 1 µg of each plasmid construct was transfected into HEK293 cells using Lipofectamine® 2000 (Thermo Fisher Scientific). A Renilla luciferase plasmid, pCMV-RL (Liu et al., 2018), was co-transfected with each construct for normalization. After a further 24-hour incubation, cells were washed with phosphate buffered saline and harvested with luciferase cell culture lysis reagent from Dual-Luciferase® Reporter assay kit (Promega). Bcl-2 promoter activity in the cells was measured with the Luciferase Reporter Assay System using a Sirius luminometer (Titertek-Berthold, Bad Wildbad, Germany). Luciferase activity was calculated in relative light units and normalized to the pCMV-RL vector containing the Renilla luciferase as control reporter.

### Quantitative ChIP assay

The ChIP assay was performed using the Agarose ChIP Kit (Thermo Fisher Scientific) according to the manufacturer’s instructions. Cells were cross-linked *in situ* by addition of 16% formaldehyde to a final concentration of 1% and incubated at room temperature for 10 min, and then were incubated with glycine for 5 min. Cells were lysed and digested by Micrococcal Nuclease provided by the kit. The samples were then incubated with ANTI-FLAG (Sigma Aldrich, St. Louis, MO, USA), PRDM10 (abcam), IgG (Thermo Fisher Scientific), RNA Polymerase II (Thermo Fisher Scientific) antibody overnight at 4 °C on a rocking platform. ProteinA/G plus Agarose were then added to each sample and incubated for 1 h before washing them with wash buffers. Samples were then treated with elution buffer, followed by treatment with NaCl and Proteinase K. DNA was then extracted from the digested samples. Extracted DNA sample (the input sample and ChIP DNA sample) was used for quantitative PCR amplification using primers specifc to promoter fragments of the Bcl-2 P1 promoter and control primers. Positive control primers were from the human β-actin gene and negative primers were from the Bcl-2 p2 promoter. Human Bcl-2 P1 promoter regions were identified using data from Ensemble (http://www.ensembl.org). They were located at human chromosome 18: 63,123,346-63,320,128. The primers for Bcl-2 P1 promoter detection were the following (product length, 107 bp): forward primer, 5′-GGCTCAGAGGAGGGCTCTTT- 3′; reverse primer, 5′-GTGCCTGTCCTCTTACTTCATTCTC- 3′ (Catz & Johnson, 2001).

### RNAseq analysis

The mRNA expression analysis was performed using the TCGABiolinks package (v. 2.10.0) of R software (R Core Team, 2018) (Colaprico et al., 2016). Harmonized expression data (hg38) were downloaded from The Cancer Genome Atlas (TCGA) using the GDCdownload function. The RNA-Seq-based expression level was normalized by the Fragments Per Kilobase of transcript per Million mapped reads upper quartile (FPKM-UQ) method. Hexagonal heatmap of 2d bin was plotted using the geom_hex function from ggplot2 package (v. 3.1.0) (Hadley). First, counts the number of cases in each hexagon, and then maps the number of cases to the hexagon fill.

### Statistical analysis

Differences between two groups affected by only one factor were analyzed by Mann–Whitney test. Statistical significance of differences between multiple groups was analyzed by using Kruskal and Wally *H* test. These tests were performed using SPSS software version 19 (IBM Corporation, Armonk, NY, USA). Statistical significance was set at *P < 0.05.

## Results

### Human PRDM10 regulates the Bcl-2 expression

**Figure 1:**
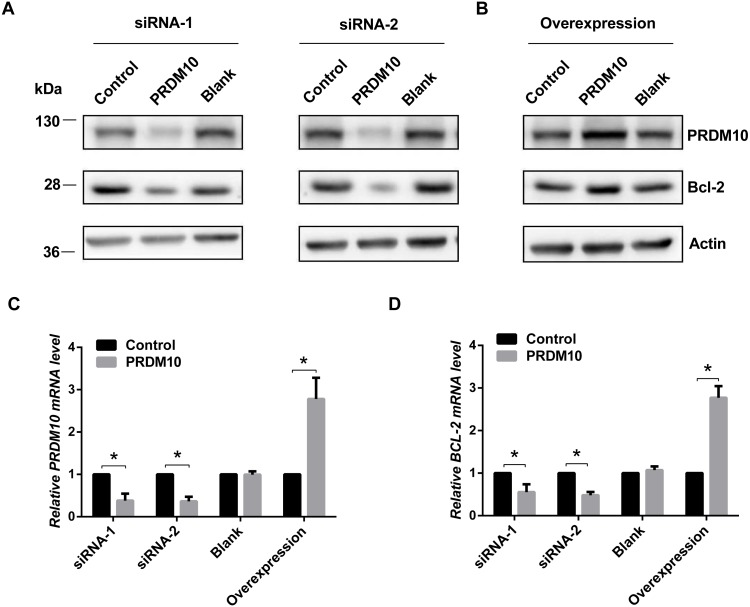
Bcl-2 expression in PRDM10-depleted and PRDM10-overexpressed cells. (A) Western blot analyses of Bcl-2 proteins in PRDM10-depleted cells (siRNA- PRDM10). (B) Bcl-2 protein levels in PRDM10 (pCMV-4A-PRDM10) transfected HEK293 cells. (C) PRDM10 mRNA levels in PRDM10-depleted or PRDM10-overexpressed cells as measured by quantitative RT-PCR. (D) Bcl-2 mRNA levels in PRDM10-depleted or PRDM10-overexpressed cells as measured by quantitative RT-PCR. The average of three independent experiments plus standard deviation is shown. *p < 0.05, *t* test, two-tailed.

We performed a candidate gene approach to search for cancer genes that might be regulated by PRDM10. By immunoblot approach, we found significant reduction (>2 fold) in protein levels for Bcl-2 and CCND1 but no changes for p53, MDM2, and BAX in PRDM10 depleted HEK293 cells as achieved by siRNA approach (Fig. S1). We focused on Bcl-2 for in depth analyses and further found that overexpression of PRDM10 was able to up regulate Bcl-2 protein expression (Figs. 1A and 1B). By quantitative reverse transcriptase-PCR analyses, we found that Bcl-2 mRNA levels were down-regulated by siRNA to PRDM10 and up regulated by PRDM10 overexpression (Figs. 1C and 1D).

**Figure 2:**
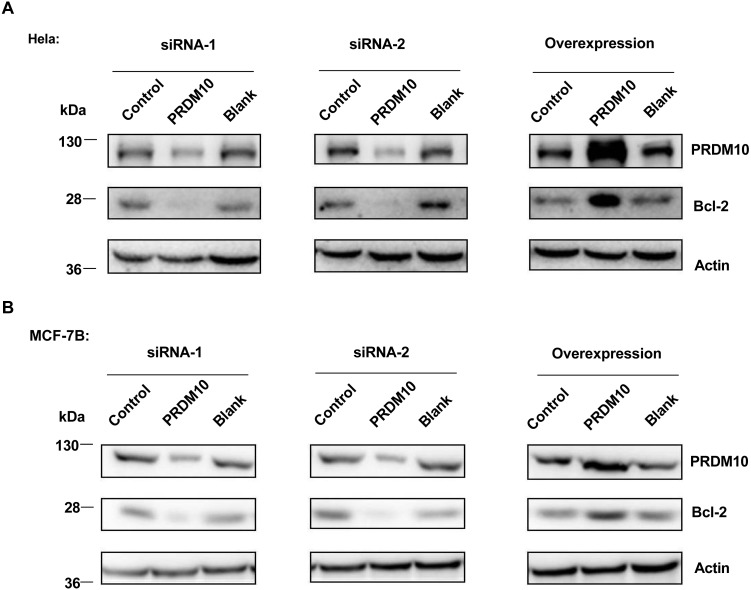
PRDM10 effect on Bcl-2 expression in different cell lines. (A) Western blot analyses of Bcl-2 proteins upon PRDM10 depletion (siRNA-PRDM10) or PRDM10-overexpression (pCMV-4A-PRDM10) in Hela cells. (B) Bcl-2 protein levels upon PRDM10-depletion or PRDM10-overexpression in MCF-7B cells.

We also tested whether PRDM10 protein contributes to Bcl-2 expression in other human cell types. We found that PRDM10 regulated Bcl-2 protein levels in Hela and MCF-7B cell lines (Fig. 2). So, the effect of PRDM10 on Bcl-2 expression did not appear to be cell type dependent.

### PRDM10 as a transcriptional activator

**Figure 3:**
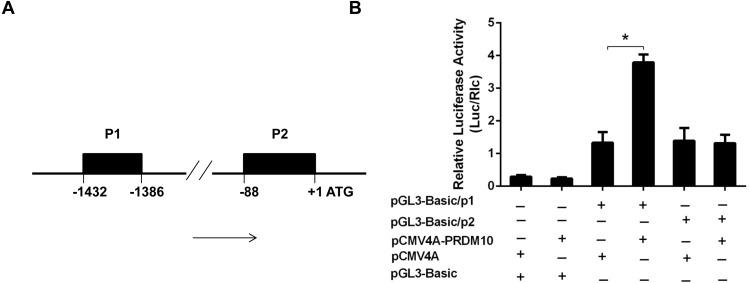
PRDM10 on Bcl-2 gene promoter activity. (A) Schematic map of human Bcl-2 gene promoter containing the 5′ (P1) promoter and 3′ (P2) promoter region. (B) Results of promoter report assay. Dual luciferase activity was measured by a luminometer at 48 h after transfection. The values represent means ± SD; *n* = 3; *p < 0.05, *t* test, two-tailed.

We next determined whether PRDM10 may regulate Bcl-2 transcription. As the P1 promoter is the major element in driving Bcl-2 transcription, we focused on this promoter in our analyses here (Petrovic et al., 1998; Young & Korsmeyer, 1993; Tsujimoto & Croce, 1986). The Bcl-2 P1 promoter reporter plasmid was generated by inserting the (−1,386 to −1,444 bp) human Bcl-2 P1 promoter sequence (Duan, Heckman & Boxer, 2007; Onel et al., 2016) in front of the firefly luciferase reporter gene as in pGL3 basic vector (Fig. 3A). The Bcl-2 p2 promoter construct was created by inserting the (−754 to +1 bp) human P2 promoter seguence (Catz & Johnson, 2001). We then carried out Dual-lucifease assays as described previously (Liu et al., 2018) on cells cotransfected with the Bcl-2 promoter construct and PRDM10 expression construct. The results showed higher luciferase activity in cells transfected with Bcl-2 P1 promoter and pCMV4A-PRDM10 (Fig. 3B). No changes were observed for the Bcl-2 p2 promoter reporter (Fig. 3B). These results suggest that PRDM10 may be a positive regulator of the Bcl-2 P1 promoter.

### PRDM10 binding to the P1 promoters of Bcl-2 *in vivo*

**Figure 4:**
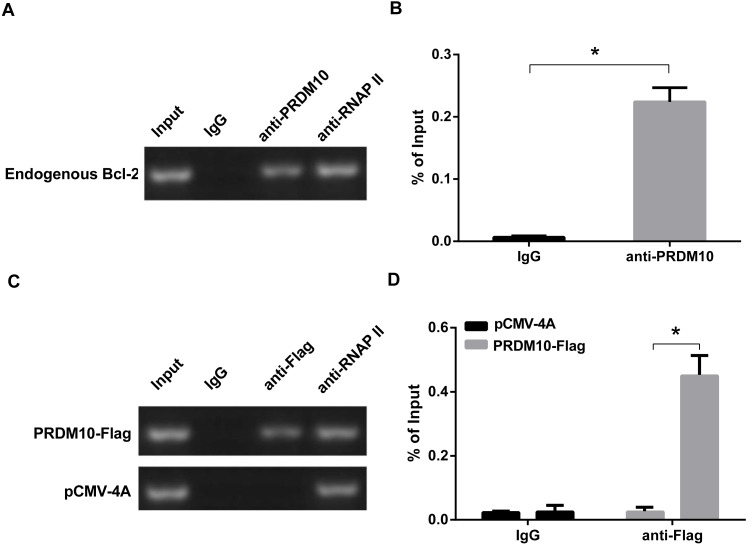
ChIP assay on Bcl-2 gene P1 promoter. (A) ChIP in endogenous HEK293 cells. PCR was performed using primers Bcl-2 P1 promoter. (B) Quantitative PCR analyses of ChIP assay of PRDM10 binding to the Bcl-2 P1 promoter in HEK293 cells. As shown, the cross-linked chromatin was precipitated with specific antibodies. The results are shown as the percentage of input DNA. (C) ChIP in transfected HEK293 cells. HEK293 cells were transfected with pCMV4A/PRDM10-Flag or pCMV-4A vectors (Negative). PCR was performed using primers Bcl-2 P1 promoter. (D) Quantitative PCR analyses of ChIP assay of PRDM10 binding to the Bcl-2 P1 promoter in transfected cells. HEK293 cells were transfected with pCMV-4A/PRDM10-Flag or pCMV-4A vectors (Negative). The values represent means ± SD; *n* = 3; *p < 0.05, *t* test, two-tailed.

As PRDM10 was found to regulate the P1 promoter of Bcl-2, we next determined whether PRDM10 could directly bind to the Bcl-2 P1 promoter. Based on human genomic sequences of Bcl-2 P1 gene available from the UCSC database, we designed PCR primers to cover these sites and performed chromatin immunoprecipitation (ChIP) assays and quantitative PCR to examine the binding of PRDM10 protein to the Bcl-2 P1 promoter in HEK293 cells. PRDM10 binding was detected in HEK293 cells (Figs. 4A and 4B) and also in PRDM10-Flag plasmid transfected cells (Figs. 4C and 4D).

### Correlation between Bcl-2 and PRDM10 expression in cancers

**Figure 5:**
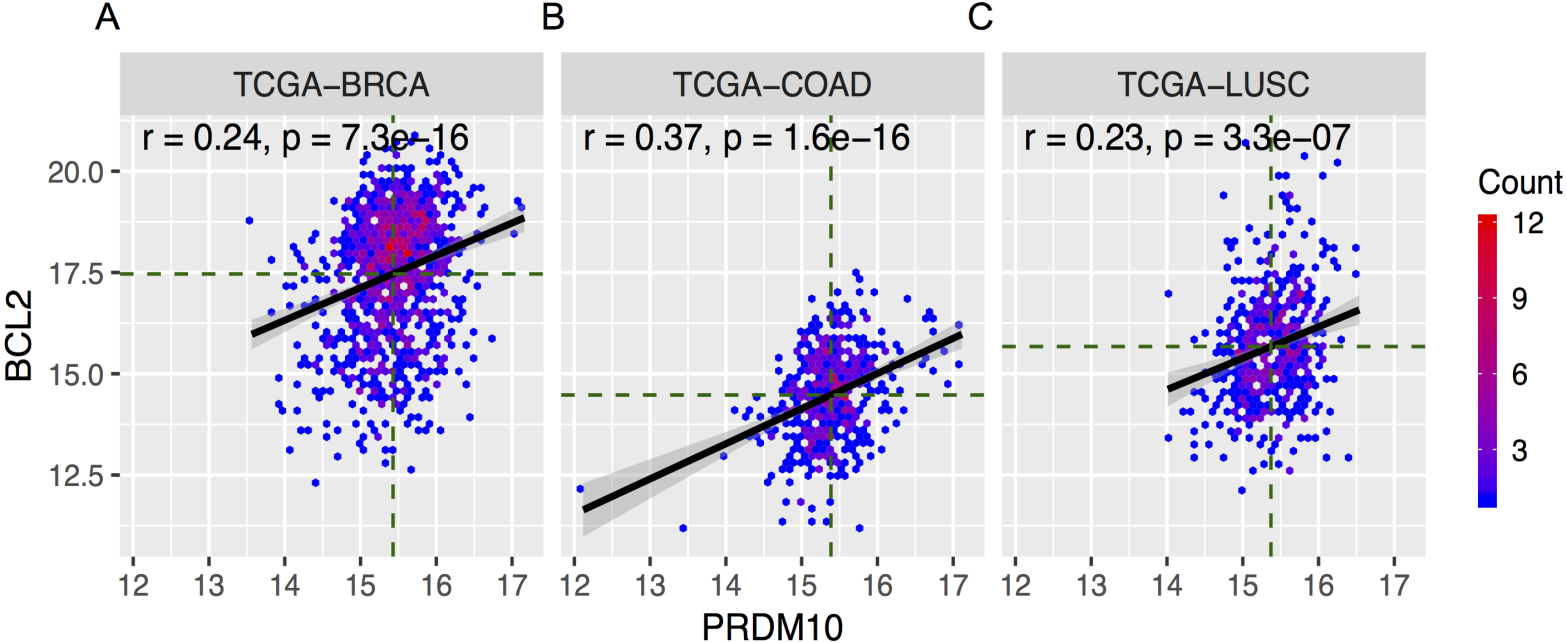
Pooled analyses on the Correlation between Bcl-2 and PRDM10 expression in cancers. Hexagonal heatmap of correlation between expression of Bcl-2 and PRDM10 in the primary solid tumor tissues of breast invasive carcinoma (BRCA, left), colon adenocarcinoma (COAD, middle), and lung squamous cell carcinoma (LUSC, right). The *x*-axis and *y*-axis represent the value of log2 FPKM. The *r* represents the value of Pearson’s correlation coefficient, *p* represents the *p*-value of test for the Pearson’s correlation coefficient.

As Bcl-2 is often over expressed in cancers, we next studied The Cancer Genome Atlas (TCGA) to see if Bcl-2 and PRDM10 expression may be correlated. We downloaded RNA seq datasets from the TCGA web portal for 31 cancer types and about 11,000 patient samples. PRDM10 has previously been found overexpressed in breast, ovary, kidney, colon, lung and prostate cancers (Sorrentino et al., 2018). Consistently, we found borderline positive correlation between Bcl-2 and PRDM10 expression in breast, colon, and lung cancers (Fig. 5 and Table 1). Bcl-2 is known to be highly expressed in follicular lymphomas but the TCGA dataset has no informative information for this tumor.

## Discussion

Here we present evidence that PRDM10 protein affected Bcl-2 gene expression at the transcriptional level. PRDM10 protein has been found overexpressed in certain cancers (Sorrentino et al., 2018) and hence may be expected to have oncogene like activities. As Bcl-2 is highly oncogenic, our study here provides a potential way for how PRDM10 may contribute to tumorigenesis. Many studies have linked the expression of Bcl-2 to the development of cancers (McDonnell et al., 1992; Lin et al., 2007; Lindner et al., 2017; Trudel et al., 1997; Yang et al., 2017). Importantly, our results showed correlation of Bcl-2 and PRDM10 expression in cancers overexpressing PRDM10. Therefore, up-regulation of Bcl-2 by PRDM10 may be a potential mechanism for tumoriginesis in cancers overexpressing PRDM10. However, the mechanisms of PRDM10 action in up-regulating gene transcription remains to be better understood.

A potential role for PRDM10 in cancer is to be highly expected since several better studied members of the PRDM family, PRDM2, PRDM3, PRDM5, and PRDM14, are known to play an important role in a variety of cancers (Huang, 2002; Emterling et al., 2004; Canote et al., 2002; Deng & Huang, 2004; Nishikawa et al., 2007). While some such as PRDM2 and PRDM5 act as tumor suppressors, PRDM14 appears to be an oncogene (Nishikawa et al., 2007). The results here suggest that PRDM10 may also play an oncogenic role in cancer.

Bcl-2 protein determines the response of cancer cells to chemotherapeutic agents. In this context, the research and development of Bcl-2 inhibitors is believed to have great potential for the discovery of novel pharmacological modulators in cancer. It was reported that ABT-199 has promising activity against preclinical models in some cancers (Sasi et al., 2019; Flinn et al., 2019; Lok et al., 2019). Results of a phase II clinical trial have shown that ABT-199 has promising clinical activity compared to current alternatives, but relapse remains a concern (Rogers et al., 2018; De Vos et al., 2018). Combinations such as ABT-199 with decitabine or azacitidine can help induce remission but lack improvement in overall survival (DiNardo et al., 2019; Rahmat et al., 2018). Thus, new combinations involving ABT-199 are urgently needed to manage cancer remission. Elucidating the regulation of the Bcl-2 expression may provide new opportunities for designing anti-Bcl-2 agents.

**Table 1:** Number of samples.

Abbr.	Cancers	*N* of samples
BRCA	Breast Invasive Carcinoma	1,102
COAD	Colon Adenocarcinoma	478
LUSC	Lung Squamous Cell Carcinoma	502

## Conclusions

We here found that PRDM10 could bind to the p1 promoter of Bcl-2 and contribute to its expression. Like its related PR domain genes, PRDM10 also displays properties of transcription factors with a potential role in tumorigenesis. Future studies will be required to determine whether siRNA targeting PRDM10 may serve as novel cancer therapy agents by inhibiting Bcl-2 expression.

##  Supplemental Information

10.7717/peerj.6941/supp-1 Supplemental Information 1 Full-length uncropped blots Figure 1. Bcl-2 expression in PRDM10-depleted and PRDM10-overexpressed cells. (A) Immuno-blot analyses of Bcl-2 proteins in PRDM10-depleted cells (siRNA- PRDM10). (B) Bcl-2 protein levels in PRDM10 (pCMV-4A-PRDM10) transfected HEK293 cells.
Figure 2. PRDM10 effect on Bcl-2 expression in different cell lines. (A) Western blot analyses of Bcl-2 proteins upon PRDM10 depletion (siRNA-PRDM10) or PRDM10-overexpression (pCMV-4A-PRDM10) in Hela cells. (B) Bcl-2 protein levels upon PRDM10-depletion or PRDM10-overexpression in MCF-7B cells.
Figure 4A ChIP in endogenous HEK293 cells. PCR was performed using primers Bcl-2 P1 promoter.C ChIP in transfected HEK293 cells.

10.7717/peerj.6941/supp-2 Dataset S1 Bcl-2 mRNA expression in PRDM10-depleted and PRDM10-overexpressed cells PRDM10 mRNA levels in PRDM10-depleted or -overexpressed cells as measured by quantitative RT-PCR. Bcl-2 mRNA levels in PRDM10-depleted or PRDM10-overexpressed cells as measured by quantitative RT-PCR

10.7717/peerj.6941/supp-3 Dataset S2 PRDM10 on** Bcl-2 gene promoter activity Results of promoter report assay

10.7717/peerj.6941/supp-4 Dataset S3 ChIP assay on Bcl-2 gene P1 promoter Quantitation of ChIP assay of PRDM10 binding to the Bcl-2 P1 promoter in HEK293 cells.
Quantitation of ChIP assay of PRDM10 binding to the Bcl-2 P1 promoter in transfected cells

10.7717/peerj.6941/supp-5 Table S1 Supplementary Materials Supplementary Table 1.Antibodies used for Western blot
Supplementary Table 2. Primers used for real-time PCR analysis

## Additional Information and Declarations

### Data Availability

The following information was supplied regarding data availability: The raw measurements are available in the Supplemental File.
