# Supplementary material for: Transcriptional regulation of Bcl-2 gene by the PR/SET domain family member PRDM10
Source: PeerJ. 2019 May 15;7:e6941. doi: 10.7717/peerj.6941 (PMC6525587; doi:10.7717/peerj.6941)
Supplement: Supplemental Information 1 — Figure 1. Bcl-2 expression in PRDM10-depleted and PRDM10-overexpressed cells. (A) Immuno-blot analyses of Bcl-2 proteins in PRDM10-depleted cells (siRNA- PRDM10). (B) Bcl-2 protein levels in PRDM10 (pCMV-4A-PRDM10) transfected HEK293 cells. Figure 2. PRDM10 effect on Bcl-2 expression in different cell lines. (A) Western blot analyses of Bcl-2 proteins upon PRDM10 depletion (siRNA-PRDM10) or PRDM10-overexpression (pCMV-4A-PRDM10) in Hela cells. (B) Bcl-2 protein levels upon PRDM10-depletion or PRDM10-overexpression in MCF-7B cells. Figure 4A ChIP in endogenous HEK293 cells. PCR was performed using primers Bcl-2 P1 promoter.C ChIP in transfected HEK293 cells. [file peerj-07-6941-s001.zip › Raw-data.pptx]

## Slide 1
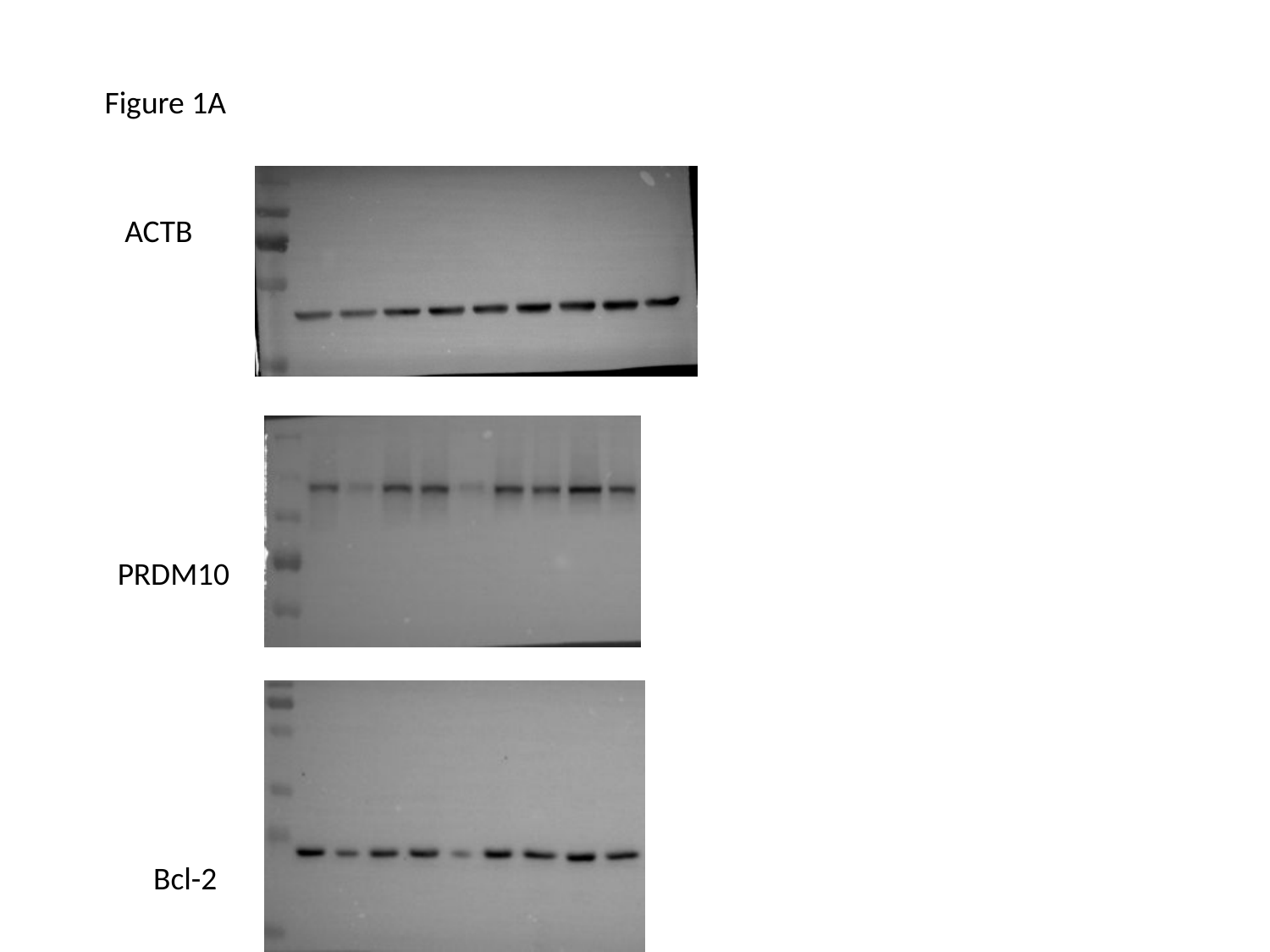

Figure 1A
 ACTB
PRDM10
 Bcl-2

## Slide 2
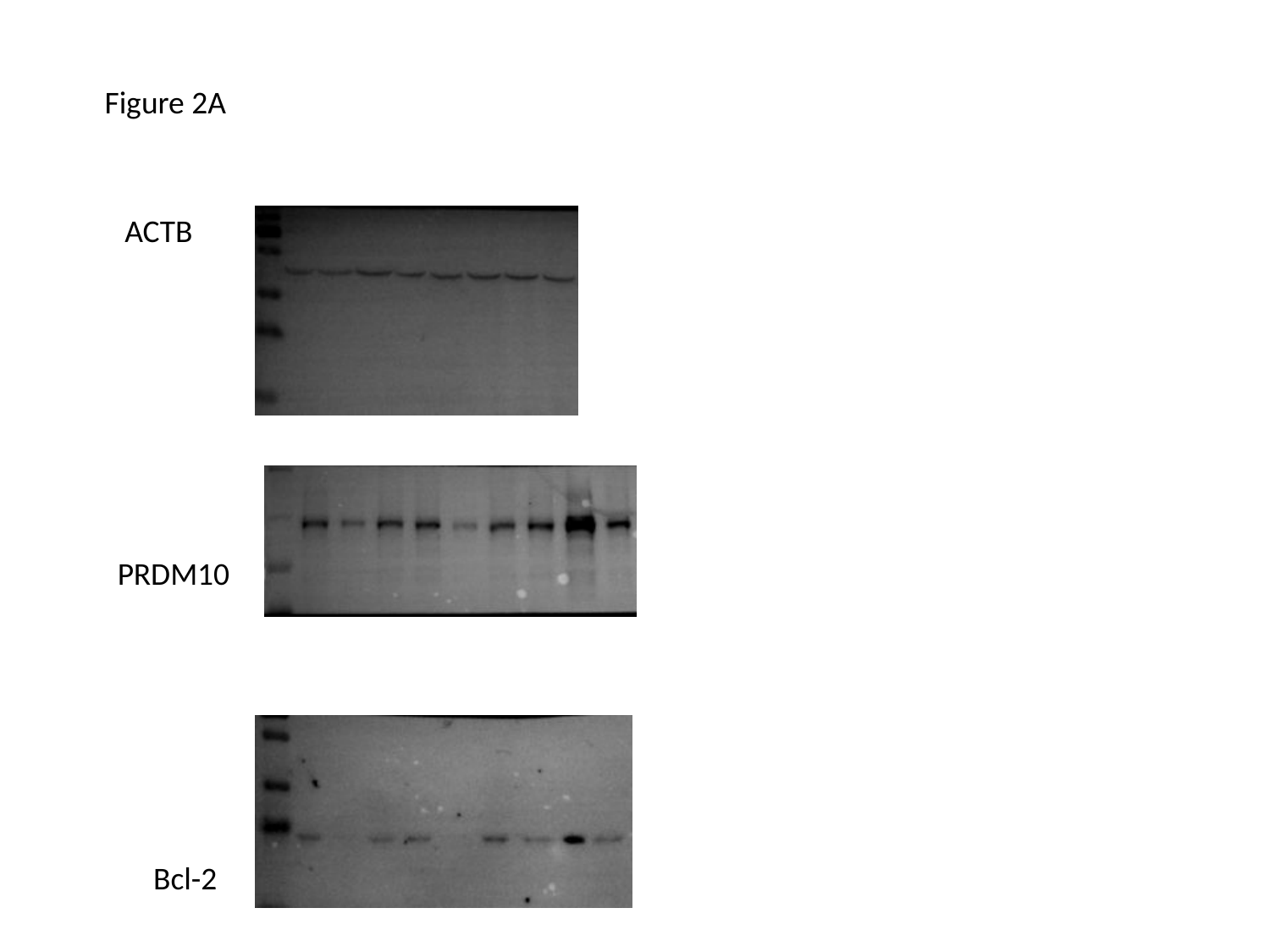

Figure 2A
 ACTB
PRDM10
 Bcl-2

## Slide 3
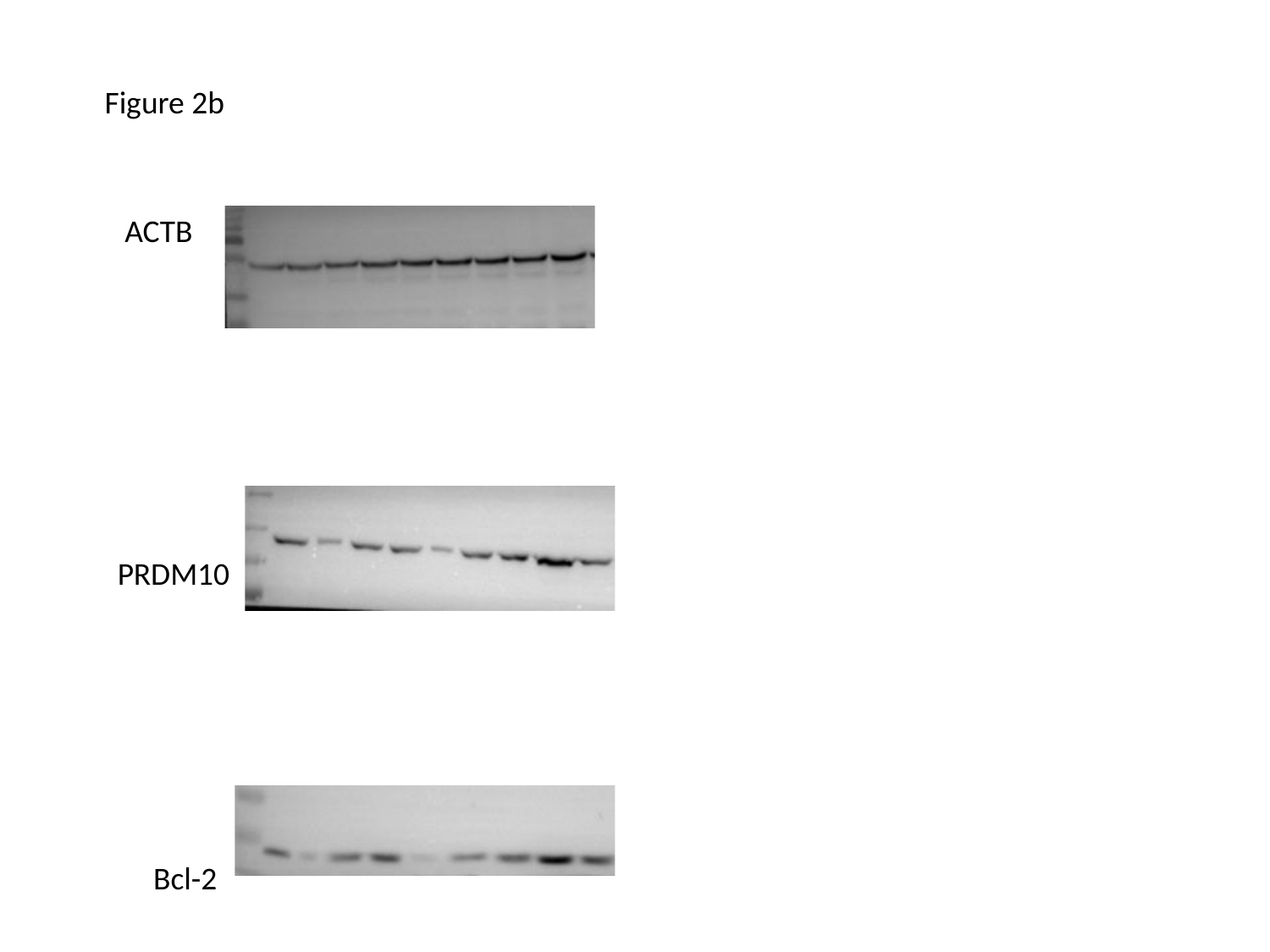

Figure 2b
 ACTB
PRDM10
 Bcl-2

## Slide 4
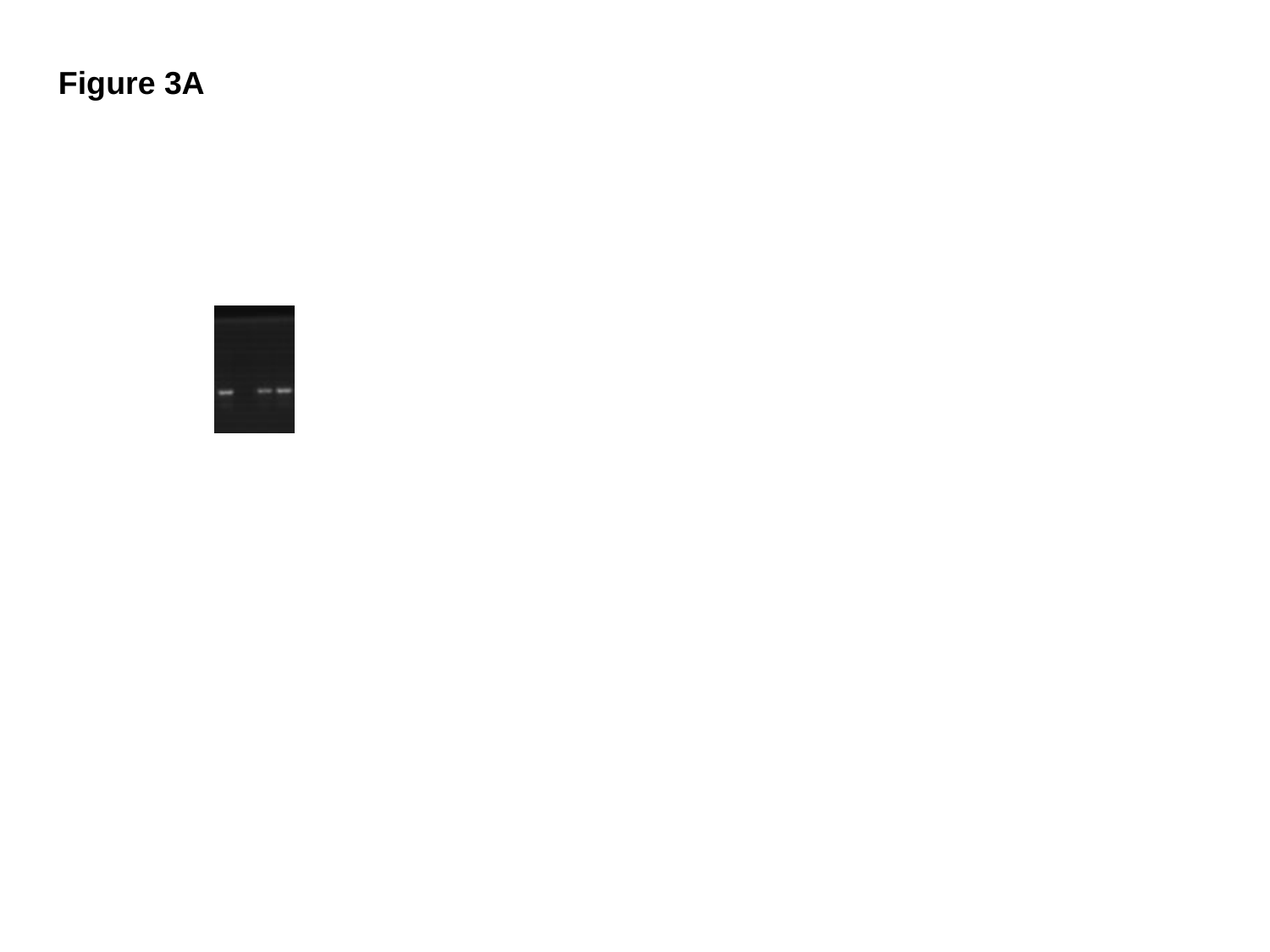

Figure 3A

## Slide 5
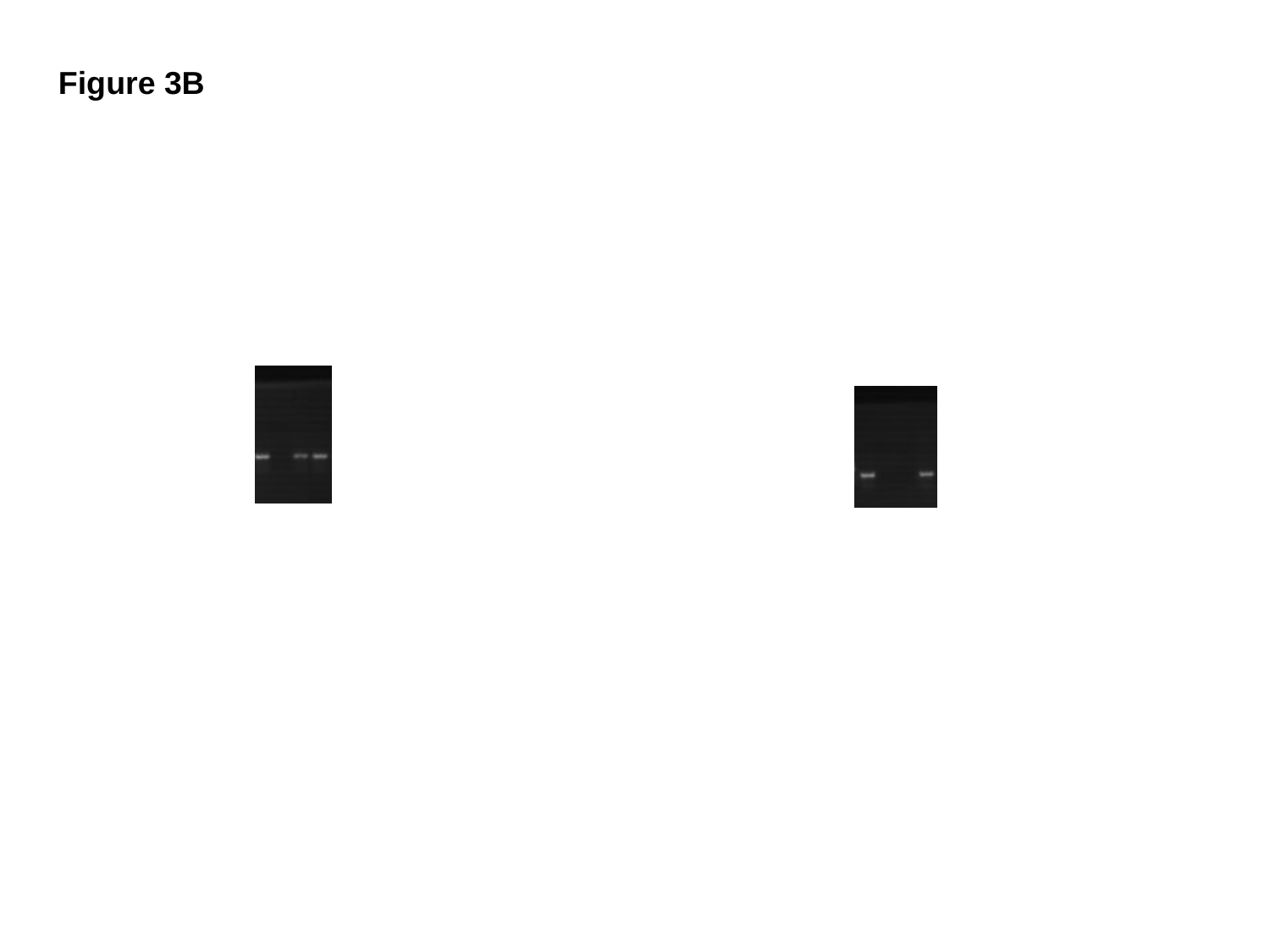

Figure 3B
