# Supplementary material for: Transcriptional regulation of Bcl-2 gene by the PR/SET domain family member PRDM10
Source: PeerJ. 2019 May 15;7:e6941. doi: 10.7717/peerj.6941 (PMC6525587; doi:10.7717/peerj.6941)
Supplement: Table S1 — Supplementary Table 1.Antibodies used for Western blot Supplementary Table 2. Primers used for real-time PCR analysis [file peerj-07-6941-s005.docx]

**Transcriptional regulation of Bcl-2 gene by the PR/SET domain family member PRDM10**

Na Chen^1^, Taobo Hu^2^, Yuanyuan Gui^1^, Jieying Gao^1^,Qingnan Liu^1^, Zhihong Li^2^, and Shi Huang^1*^

^1^Center for Medical Genetics, School of Life Sciences, Central South University, Changsha, Hunan, China 410078

^2^ Department of Orthopedics, the Second Xiangya Hospital, Central South University, Changsha, China 410011

**Supplementary Figure 1**

**
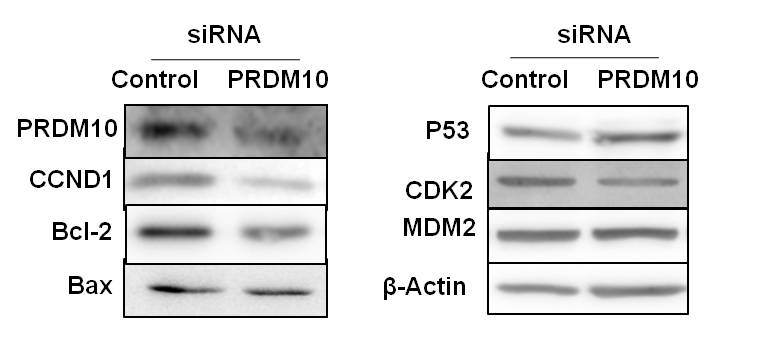
**

**Figure 1 cancer Related genes expression in PRDM10-depleted cells.** Immuno-blot analyses of Bcl-2 proteins in PRDM10-depleted cells (siRNA- PRDM10).

**Supplementary Materials**

**Supplementary Table 1.Antibodies used for Western blot**

| Protein | Catalog Number | MW(kDa) | manufacturer | Type |
| --- | --- | --- | --- | --- |
| β-actin | D110001 | 42 | Sangon | Rabbit PAb |
| PRDM10 | Ab3787 | 120 | Abcam | Mouse MAb |
| Bcl-2 | SC-509 | 28 | SantaCruz | Mouse MAb |
| BCl-2 | D160117 | 28 | Sangon | Rabbit PAb |
| Flag | F1804 | - | SIGMA | Mouse MAb |
| IgG | D110502 | - | Sangon | Rabbit PAb |

**Supplementary Table 2. Primers used for real-time PCR analysis**

| qRT-PCR-analysis | sequence | Product length(bp) |
| --- | --- | --- |
| β-actin | FP^1^: AGCGAGCATCCCCCAAAGTT  RP^2^: GGGCACGAAGGCTCATCATT | 285 |
| PRDM10 | FP: GCTGCCTTCCATCGAGAGTG  RP: CCAGTCATCCAGATCCGTGTC | 160 |
| Bcl-2 | FP: GGTGGGGTCATGTGTGTGG  RP: CGGTTCAGGTACTCAGTCATCC | 89 |

FP^1^: Forward Primer

RP^2^: Reverse Primer
